# Macrophage Polarization in Physiological and Pathological Pregnancy

**DOI:** 10.3389/fimmu.2019.00792

**Published:** 2019-04-15

**Authors:** Yongli Yao, Xiang-Hong Xu, Liping Jin

**Affiliations:** Clinical and Translational Research Center, Shanghai First Maternity and Infant Hospital, Tongji University School of Medicine, Shanghai, China

**Keywords:** macrophage, maternal-fetal interface, polarization, abortion, preeclampsia, preterm birth

## Abstract

The immunology of pregnancy is complex and poorly defined. During the complex process of pregnancy, macrophages secrete many cytokines/chemokines and play pivotal roles in the maintenance of maternal-fetal tolerance. Here, we summarized the current knowledge of macrophage polarization and the mechanisms involved in physiological or pathological pregnancy processes, including miscarriage, preeclampsia, and preterm birth. Although current evidence provides a compelling argument that macrophages are important in pregnancy, our understanding of the roles and mechanisms of macrophages in pregnancy is still rudimentary. Since macrophages exhibit functional plasticity, they may be ideal targets for therapeutic manipulation during pathological pregnancy. Additional studies are needed to better define the functions and mechanisms of various macrophage subsets in both normal and pathological pregnancy.

## Introduction

At the maternal-fetal interface, macrophages are the second largest group of cells and comprise 20–30% of all leukocytes (1). These cells display important roles in the pregnancy process as their plastic characteristics. Plastic characteristics refer to macrophage polarization, through which macrophages differentiate into specific phenotypes and have specific biological functions in response to microenvironmental stimuli. By simplified classification, macrophages have been divided into M1 and M2 subtypes based on their activation states (2). Actually, the properties of M1-like/M2-like macrophages are similar to those of Th1/Th2 cells (3). M1 macrophages are functionally pro-inflammatory and antimicrobial, while M2 macrophages are anti-inflammatory (4, 5).

At the maternal-fetal interface, both the number and proportion of M1/M2 macrophages are changed during different gestation periods to protect the fetus from the maternal immune microenvironment and establish foetomaternal tolerance. To sustain foetomaternal tolerance, more macrophages are polarized into alternatively activated (M2-like) macrophages, implying that the immunosuppressive properties of M2 macrophages are necessary for normal pregnancy. In abnormal pregnancy, more classically activated (M1) macrophages have been observed at the maternal-fetal interface. The balance of polarization between M1 and M2 macrophages is important for various processes of normal pregnancy, such as trophoblast invasion, spiral artery remodeling, and apoptotic cell phagocytosis. Conversely, the dysregulated polarization of macrophages was associated with inadequate remodeling of the uterine vessels and defective trophoblast invasion and finally led to spontaneous abortion, preeclampsia and preterm birth (6–8).

Although increasing evidence has indicated the critical roles of macrophages in pregnancy-related diseases, the molecular mechanisms of dysregulated macrophage polarization are still poorly understood. Here, we summarize the current knowledge of macrophage polarization and the mechanisms involved in physiological or pathological pregnancy processes. A deeper understanding of the roles of macrophages in gestation might allow us to develop therapies to improve pregnancy outcomes.

## The Polarization of Macrophages

### M1 and M2 Macrophages

Macrophage polarization is crucial for tissue repairing and homeostasis maintenance (9). Macrophage polarization refers to the process by which macrophages produce distinct functional phenotypes as a reaction to specific microenvironmental stimuli and signals (3, 10–12). Macrophages can be polarized into classically activated (M1) and alternatively activated (M2) macrophages. M2 macrophages are divided into M2a, M2b, M2c, and M2d subcategories. These macrophages differ in their cell surface markers, secreted cytokines and biological functions. However, studies have indicated that the induction routes and regulated biological processes are complex interlacing network systems rather than simplistic schema (13). M1/M2 polarity arises from arginine metabolism via two antagonistic pathways: M1-like macrophages are the products of the iNOS pathway, which produces citrulline and NO from arginine, whereas M2-like macrophages are the products of the arginase pathway, which produces ornithine and urea from arginine (14).

Following the activation by lipopolysaccharide (LPS) and Th1 cytokines (such as IFN-γ and TNF-α), macrophages are polarized into M1 macrophages and characterized by TLR-2, TLR-4, CD80, CD86, iNOS, and MHC-II surface phenotypes. These cells release various cytokines and chemokines (for example, TNF-α, IL-1α, IL-1β, IL-6, IL-12, CXCL9, and CXCL10) which exert positive feedback on unpolarized macrophages. That is, these factors attract more unpolarized macrophages to M1 state. Key transcription factors, such as NF-kB, STAT1, STAT5, IRF3, and IRF5 have been shown to regulate the expression of M1 genes. It seems that NF-κB and STAT1 are the two major pathways involved in M1 macrophage polarization and result in microbicidal and tumouricidal functions (2, 4, 5, 15, 16).

**Figure 1:**
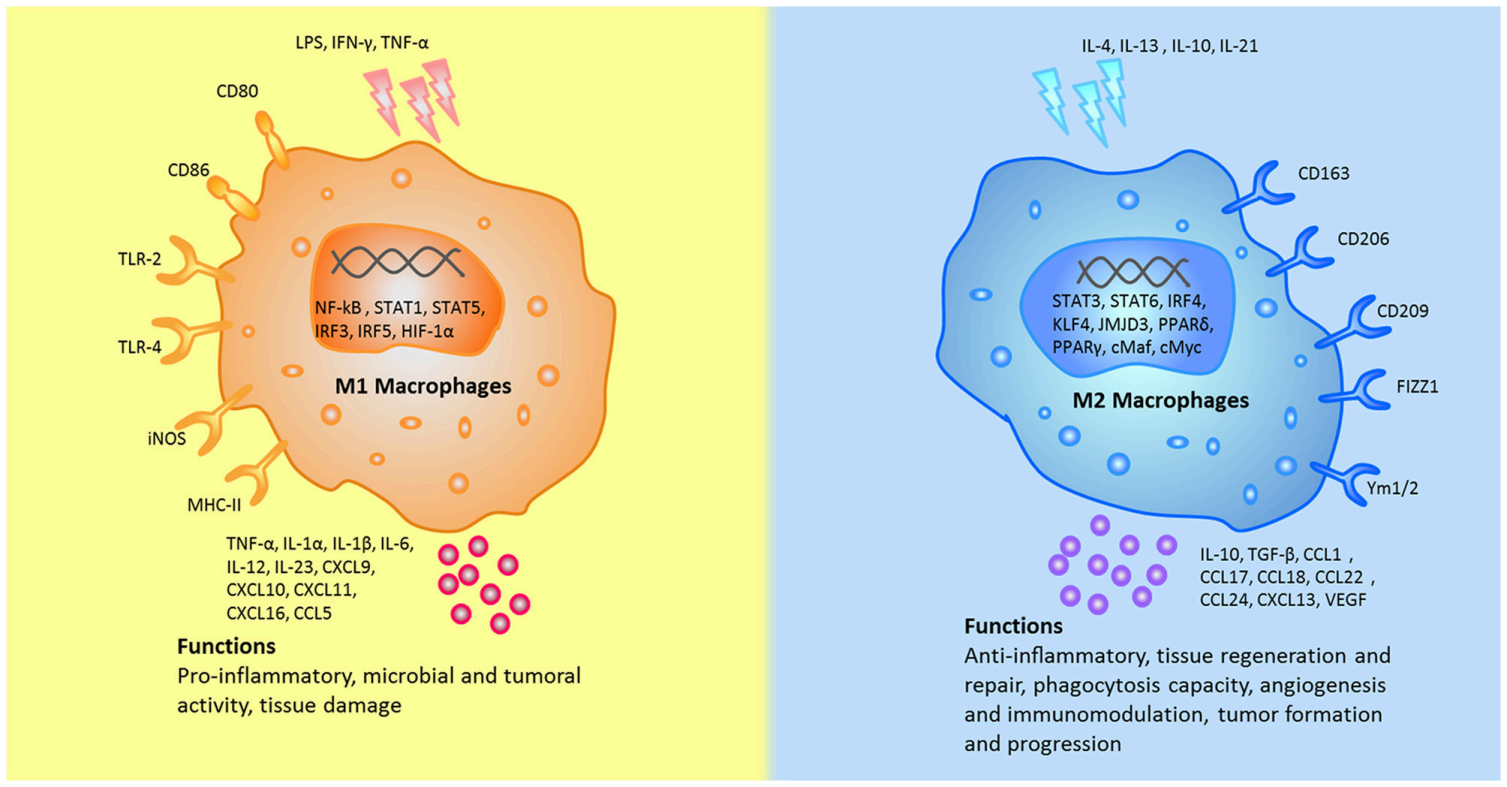
The different stumili, surface markers, secreted cytokines, and biological functions between M1 and M2 macrophages were summarized. CCL, chemokine (C-C motif) ligand; cMaf, c-musculoaponeurotic fibrosarconna; CXCL, chemokine (C-X-C) ligand; FIZZ1, resistin-like α; HIF, hypoxia inducible factor; iNOS, inducible nitric oxide synthase; IFN-γ, interferon-gamma; IL, interleukin; IRF, interferon regulatory factor; JMJD, Jumonji doman-containing protein; KLF, Kruppel-like factor; NF-κB, nuclear factor κB; KLF, Kruppel-like factor; LPS, lipopolysaccharides; MHC, major histocompatibility complex; PPAR, peroxisome proliferator-activated receptors; STAT, signal transducer and activator of transcription; TLR, Toll-like receptor; TNF-α, tumor necrosis factor alpha; TGF-β, transforming growth factor beta; VEGF, vascular endothelial growth factor; Ym1, chitinase 3-like 3.

M2 polarization occurs in response to downstream signals of cytokines such as IL-4, IL-13, IL-10, IL-33, and TGF-β (5, 16). Notably, only IL-4 and IL-13 directly induce M2 macrophage activation, whereas other cytokines (such as IL-33 and IL-25) amplify M2 macrophage activation by producing Th2 cytokines (17). M2 macrophages can be additionally identified by their expression of surface markers, such as mannitol receptor, CD206, CD163, CD209, FIZZ1, and Ym1/2. Up-regulation of cytokines and chemokines, such as IL-10, TGF-β, CCL1, CCL17, CCL18, CCL22, and CCL24 (16, 18) also attract unpolarized macrophages to polarize into the M2 state (19). Key transcription factors, such as STAT6, IRF4, JMJD3, PPARδ, and PPARγ have been shown to regulate the expression of M2 genes. Thus far, the STAT6 pathway has been considered to be the pathway to activate M2 macrophages (2). Macrophages contribute to the process of infection prevention, tissue repairing, angiogenesis and immunomodulation (5, 20). The main differences between M1 and M2 macrophages were shown in Figure 1.

### The Subsets of M2 Macrophages and Their Characteristics

**Figure 2:**
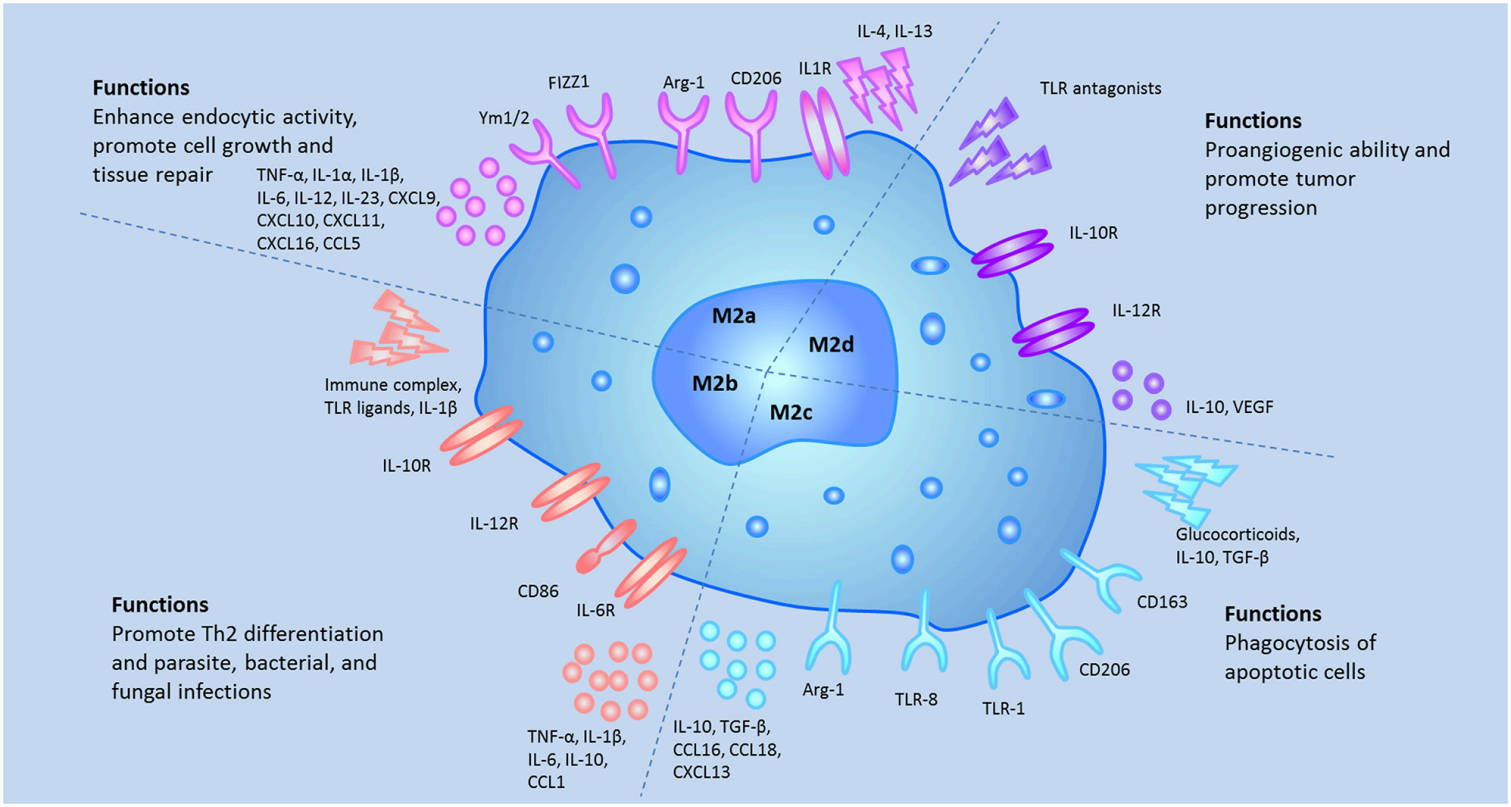
The different stumili, surface markers, secreted cytokines, and biological functions of the M2 macrophage subsets were summarized.

As mentioned above, M2 macrophages are subgrouped into M2a, M2b, M2c, and M2d. Activated by IL-4 or IL-13, M2a macrophages lead to the increased expression of IL-10, TGF-β, CCL17, CCL18, and CCL22. These macrophages enhance the endocytic activity, promote cell growth and tissue repairing. M2b macrophages are activated by immune complex, Toll-like receptor (TLR) ligands and IL-1β and release both pro- and anti-inflammatory cytokines, such as TNF-α, IL-1β, IL-6, and IL-10. Based on the expression profiles of cytokines and chemokines, M2b macrophages regulate the breadth and depth of immune responses and inflammatory reactions (21). M2c macrophages, also known as inactivated macrophages, are induced by glucocorticoids, IL-10 and TGF-β. These cells secrete IL-10, TGF-β, CCL16, and CCL18 and play crucial roles in the phagocytosis of apoptotic cells process (12, 22). Induced by the TLR antagonists, M2d macrophages lead to the release of IL-10 and vascular endothelial growth factors (VEGF) and promote angiogenesis and tumor progression (23). The characteristics of the M2 subtypes were summarized in Figure 2.

### The Common and Different Characteristics Between Macrophages and Dendritic Cells (DCs)

Although both macrophages and DCs are members of the mononuclear phagocyte system, these cells are often considered distinct cell types based on their morphology and functions. Macrophages are defined as large vacuolar cells that are highly phagocytic and modulate immune responses by releasing various immune mediators, while DC are characterized as stellate migratory cells that act as sentinels in non-lymphoid tissues and migrate into lymphoid tissues upon antigen encounter, present antigen, and activate native T lymphocytes subsequently (24–26).

**Figure 3:**
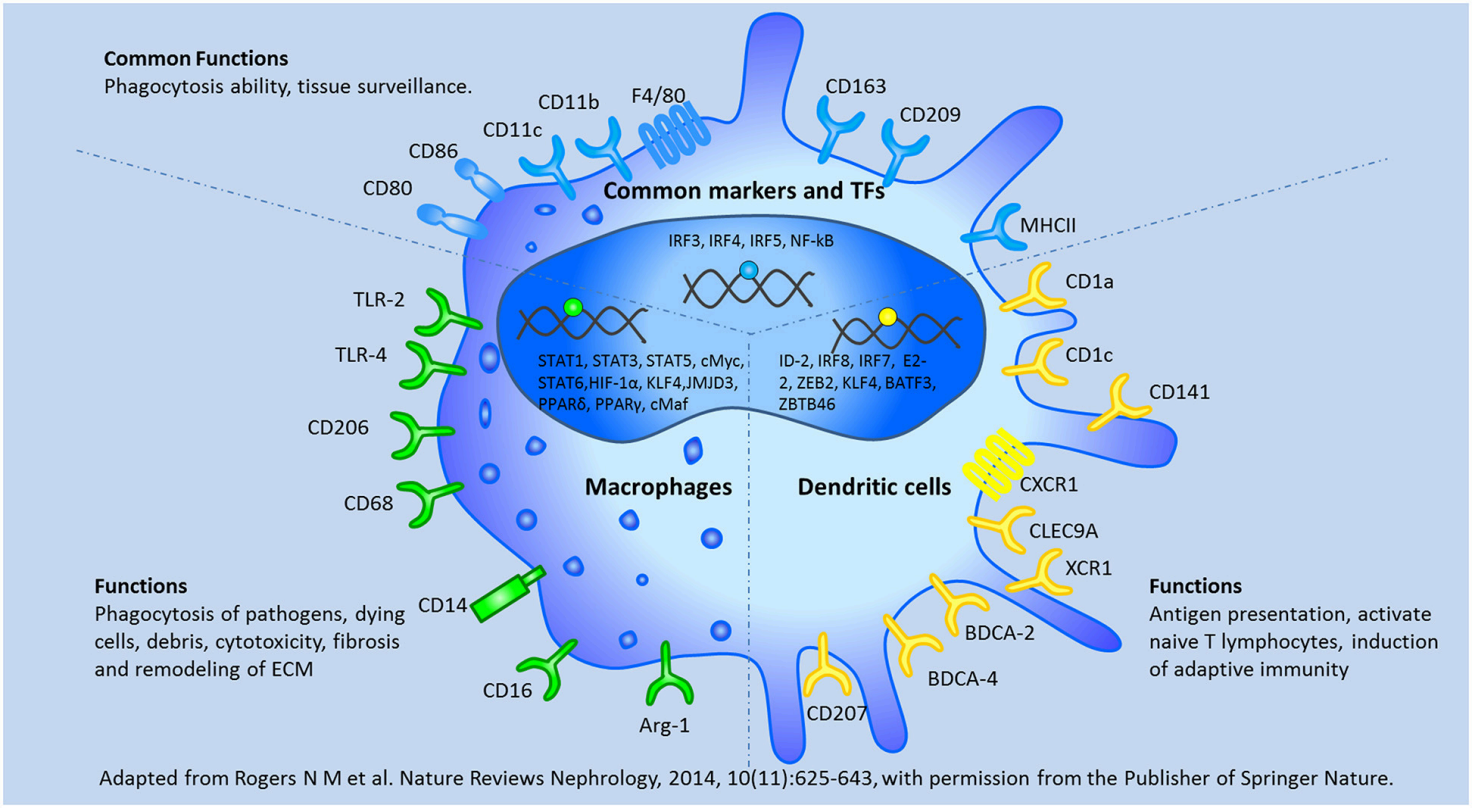
The common and different characteristics between macrophages and dendritic cells were depicted according to their surface markers, transcription factors, and biological functions. BATF3, basic leucine zipper ATF-like transcription; BDCA, blood dendritic cells Ags; CLEC9A, C-type lectin 9A; E2-2, basic helix-loop-helix transcription factor; ID-2, inhibitor of DNA binding 2; XCR1, chemokine XC receptor 1; ZBTB46, zinc finger and BTB domain containing 46; ZEB2, Zinc finger E box–binding homeobox 2. Adapted from Rogers et al. (138) with permission from the publisher of Springer Nature.

*In vitro*, macrophage colony-stimulating factor (M-CSF) induces the differentiation of monocytes into macrophages (27), while the combination of granulocyte/macrophage colony-stimulating factor (GM-CSF) and interleukin 4 (IL4) induces the differentiation of monocytes into DCs (28). Macrophages are classified into 2 subgroups (M1 and M2 [M2a, M2b, M2c, M2d]) depending on their anti- or pro-inflammatory properties (29). DCs comprise two functionally distinct populations: plasmacytoid (pDC) and myeloid (mDC). mDCs have been further subdivided into 2 subsets based on their expression of BDCA3/CD141 (mDC1) and BDCA1/CD1c (mDC2) (30). Although macrophages and DCs originate from a common myeloid precursor, these cells are distinct cell types with individual and specific transcriptional profiles (29, 31–33). Of all the different cell characteristics, surface markers are often used to distinguish DCs from macrophages, but phenotypic analysis has considered as insufficient to define DC subsets, as there are some common phenotypic markers of the cells, such as F4/80, CD11b, CD11c, CD80, CD 86, CD163, CD209, and MHCII (34). These unspecific markers may result in the misinterpretation of DCs and macrophages. Here, we summarized the common and different characteristics between DCs and macrophages in Figure 3.

## The Roles of Macrophages in Normal Pregnancy

### The Roles of M1 and M2 Macrophages in Normal Pregnancy

Macrophages comprise approximately 20–30% of all human decidual leukocytes during pregnancy (35). A study on the classification of maternal-fetal macrophages was first performed in the Stein laboratory (36). Mills et al. divided macrophages into M1 and M2 subsets based on the consistency of the Th1/Th2 immune response and their pro-inflammation or anti-inflammation properties (37). Houser et al. divided decidual macrophages into CD11c^high^ and CD11c^low^ subsets because the genes expressed in CD11c^high^ decidual macrophages are associated with lipid metabolism and inflammation, whereas the genes expressed in CD11c^low^ decidual macrophages are associated with extracellular matrix formation, muscle regulation, and tissue growth (38). In addition, some researchers have also categorized macrophages as CD209^high^ and CD209^−^ macrophages based on their differential CD209 expression levels (39). GM-CSF and M-CSF are two members of the CSF family, and these factors induce macrophages to polarize into M1-like and M2-like macrophages, respectively (14).

The polarization patterns of decidual macrophages vary with gestational age. During the peri-implantation period of gestation, macrophages polarize into M1 macrophages based on the fact that the ratio of M1/M2 macrophages in the uterus was 1.6 on the 1st day and 1.45 on the 4th day after female mice were inseminated. The a2V (a2 isoform of V-ATPase)-mediated induction of CCL2 (MCP-1), which is a macrophage chemoattractant, promoted the recruitment of M1-like macrophages during the peri-implantation period (40). When trophoblasts attach to the endometrial lining and invade the uterine stroma, decidual macrophages begin to transform to mixed M1/M2 profiles, and these mixed M1/M2 polarization patterns remain until mid-pregnancy. These macrophages are involved in extensive remodeling of the uterine vasculature, which is required to supply adequate placental–fetal blood (40, 41). After the placental development is completed, decidual macrophages shift toward a predominantly M2 phenotype, which promotes maternal immune tolerance to semiallogenic fetuses and protects fetal growth until parturition (39, 42). Although decidual macrophages show higher expression of M2 markers, such as CD206, CD163, and dendritic cell-specific ICAM-grabbing non-integrin (DC-sign) (39, 43, 44), these cells do not seem to be typical M2 macrophages, as they are induced by M-CSF and IL-10 rather than by Th2 cytokines, such as IL4 (39). At term labor, the number of M1 decidual macrophages is higher than that at term without labor (45), which suggests that pro-inflammatory macrophages may play important roles in the onset of term labor. Term decidual macrophages are involved in the initiation of labor because these cells have higher expression of CD80, CD86, CD83, HLA-DR, and CD16 and secrete higher IL-12 and lower IL-10 and TGF-β levels than decidual macrophages from mid-pregnancy (46). A previous study observed that the concentration of IL-6 was significantly higher than that of IL-10 in placental macrophages at term pregnancy (47). This observation is consistent with the findings of Osman I et al., showing that the mRNA expression of IL-6 is significantly higher during spontaneous labor in myometrium and cervical tissues than that in non-laboring tissues (48). Recently, some researchers have proposed a hypothetical model for labor. In this model, the increased levels of circulating cell-free fetal DNA activate the innate immune system by stimulating pattern-recognition receptor 9 (TLR9) in maternal macrophages and releasing a number of inflammatory cytokines, eventually triggering parturition (49).

### The Regulation Mechanisms of Macrophages in Normal Pregnancy

During pregnancy, macrophages exist in the maternal-fetal interface. Macrophages play a positive role in embryo implantation, placental formation, embryonic development, and delivery processes. In all stages of pregnancy, the maternal uterus provides a microenvironment for embryo growth by producing various cytokines, promoting trophoblast cell invasion, remodeling of spiral arteries and phagocytose apoptotic cells (50–53) (Table 1).

**Table 1:** The regulation mechanisms of macrophages in normal pregnancy.

	**Samples**	**Mechanisms**	**References**
Trophoblast invasion	Human	Decidual macrophages can inhibit NK cell-mediated lysis of CTB via TGF-β1 secretion;	(51)
	Human	IL-1β facilitates trophoblast invasion by degrading the extracellular matrix, the enzymatic activity of MMP-2, 9 is positively correlated with the level of IL-1β;	(54, 55)
	Human	sHLAG5-polarized macrophages promote the secretion of IL-6 and C-X-C motif ligand 1 to induce trophoblast invasion;	(56)
Angiogenesis and spiral artery remodeling	Human	Decidual macrophages regulate vascular remodeling by secreting VEGF, PlGF, Flt-1;	(57, 58)
	Mice	The expression of iNOS and VEGF is higher;	(59)
	Human	The sFlt-1 inhibits angiogenesis;	(60)
	THP1 cell line	VEGF promotes macrophages polarization into the M2 phenotype;	(61)
	RAW264.7 cell line	PKC inhibitor enhances the VEGF secretion and decreases the sFlt-1 secretion;	(62)
	RAW264.7 cell line and human	PSG1 upregulates the VEGFA secretion;	(63)
	Mice	PSG22 upregulates the VEGFA secretion;	(64)
Phagocytose apoptotic cells	Human	IL-12, p70, IL-1β, IL-8 are decreased, whereas IL-10, IL6, IL1Ra, IDO are upregulated;	(65, 66)
	Human	Fractalkine and calreticulin are increased in VSMCs;	(67, 68)
	Human	TGFβ induces monocyte differentiation into M2-like macrophages and enhances the capacity of phagocytosis;	(69)
	Human	sHLAG5-induced macrophages polarize into an M2 phenotype with enhanced phagocytic activity;	(56)
	Mice and RAW264.7 cell line	Tim-3 blocking antibodies cause macrophages failed to phagocytose apoptotic and dying cells;	(70)
	Human	Decidual macrophages secrete IL-1β and TNF-α to induce M-CSF expression, which initiates caspase-dependent EVT apoptosis.	(71)

#### Trophoblast Invasion

It was reported that decidual macrophages can inhibit NK cell-mediated lysis of human cytotrophoblasts (CTB) via TGF-β1 secretion (51). IL-1β secreted by activated macrophages facilitates trophoblast invasion by degrading the extracellular matrix. It has been shown that the enzymatic activity of matrix metalloproteinase (MMP)-9 and MMP-2 in trophoblastic cells is positively correlated with the concentration of IL-1β (54, 55). Immunoglobulin-like transcription factor inhibitory receptors, such as ILT2 and ILT4, can bind to HLA-G, which is highly expressed in extravillous trophoblast cells (EVT) (72). Recent work has also revealed that sHLAG5 can reduce the expression of CD86 and increase the expression of CD163. sHLAG5-polarized macrophages promote the secretion of IL-6 and C-X-C motif ligand 1, which induce trophoblast invasion (56).

#### Angiogenesis and Spiral Artery Remodeling

Angiogenesis and spiral artery remodeling of the decidua are essential to ensure sufficient blood flow to the uterus and placenta during healthy pregnancy. A previous study has shown that macrophages are involved in the early stages of the decidual spiral artery remodeling process (67). It was reported that decidual macrophages regulate vascular remodeling by secreting vascular endothelial growth factor (VEGF), placental growth factor (PlGF) and their receptors fms-like tyrosine kinase (Flt-1) (57, 58). During the embryo implantation window, the expression of iNOS and VEGF in the endometrium of pregnant mice was significantly higher than that in pseudopregnant mice. The number of macrophages was correlated with the expression levels of iNOS and VEGF in the endometrium, implying that macrophages may be involved in vascular bed development before implantation by regulating the expression of iNOS and VEGF (59). Soluble fms-like tyrosine kinase-1 (sFlt-1) is a VEGF antagonist that inhibits angiogenesis (60). An *in vivo* study showed that the macrophage M2 phenotype has a higher angiogenic potential than other macrophage subsets in C57BL/6 J mice (73). Phosphorylation of protein kinase C (PKC) is necessary for the induced expression of VEGF in various cells (74, 75). GF109203X (a general PKC inhibitor) significantly decreased LPS-induced sFlt-1 secretion and significantly enhanced LPS-induced VEGF secretion in the murine macrophage RAW264.7 cell line compared with the LPS-only treated group (62). Pregnancy-specific glycoproteins (PSG) are members of the carcinoembryonic antigen family of immunoglobulin-like genes. They are highly homologous proteins secreted by the placenta and are the most abundant fetal proteins in the maternal blood at the end of pregnancy (76, 77). Ha et al. demonstrated that the expression levels of VEGFA were upregulated by PSG1 (the most abundant member of the human PSG family) both in a mouse macrophage cell line (RAW 264.7) and in human macrophages derived from blood monocytes (63). PSG22 (the most abundant PSG expressed during mouse early pregnancy) was found to upregulate the expression of VEGFA in mouse uterine macrophages (64). These findings suggest that PSG family members in macrophages may play important roles in vascular modifications. Further studies should elucidate the exact mechanisms for M2-induced angiogenesis.

#### Phagocytose Apoptotic Cells

Macrophages phagocytose apoptotic cells to promote trophoblast invasion and spiral artery remodeling and provide a balanced microenvironment at the maternal-fetal interface during the process of pregnancy (78). It has been proposed that apoptotic cells have immunosuppressive effects (79). When trophoblast debris were phagocytosed, the levels of pro-inflammatory cytokines, such as IL-12, p70, IL-1β and IL-8, were significantly decreased, whereas the anti-inflammatory cytokines, such as IL-10, IL6, IL1Ra and IDO, were upregulated in macrophages (65, 66). Moreover, the decidual macrophages phagocytose vascular smooth muscle cells (VSMCs) to participate in spiral artery remodeling based on the fact that the expression levels of fractalkine and calreticulin were increased dramatically in VSMCs undergoing apoptosis (67, 68). Trophoblast-secreted factors, such as TGF-β, induce monocyte differentiation into M2-like macrophages and enhance the capacity of phagocytosis (69). sHLAG5-induced macrophages have also been shown to be polarized into an M2 phenotype with enhanced phagocytic activity (56). T-cell immunoglobulin and mucin domain protein 3 (Tim-3) is constitutively expressed on macrophages and is a receptor specialized for phosphatidylserine exposed on the surface of apoptotic cells (80). Treating pregnant mice with Tim-3 blocking antibodies caused the failure of uterine macrophages in mice to phagocytose apoptotic and dying cells. Thus, Tim-3 was considered to play a significant role in the process of phagocytose apoptotic cells and dying cells by macrophages (70). Decidual macrophages secrete various pro-infammatory cytokines (such as IL-1β and TNF-α) to induce M-CSF expression, which initiates caspase-dependent EVT apoptosis (71).

### The Roles of “Hofbauer Cells” in Normal Pregnancy

Hofbauer cells refers to fetal placental macrophages within the chorionic villi (81). Hofbauer cells have different origins at different stages of pregnancy. At the early stages of pregnancy, Hofbauer cells may originate from villous mesenchymal stem stromal cells or monocyte progenitor cells from the hypoblast-derived yolk sac; at later stages of pregnancy, Hofbauer cells may originate from fetal haematopoietic stem cells (82–84). Yolk sac-derived macrophages may participate in the tissue development and morphogenesis processes, while haematopoietic stem cell-derived macrophages may be important for haematopoiesis and antigen presentation processes.

**Figure 4:**
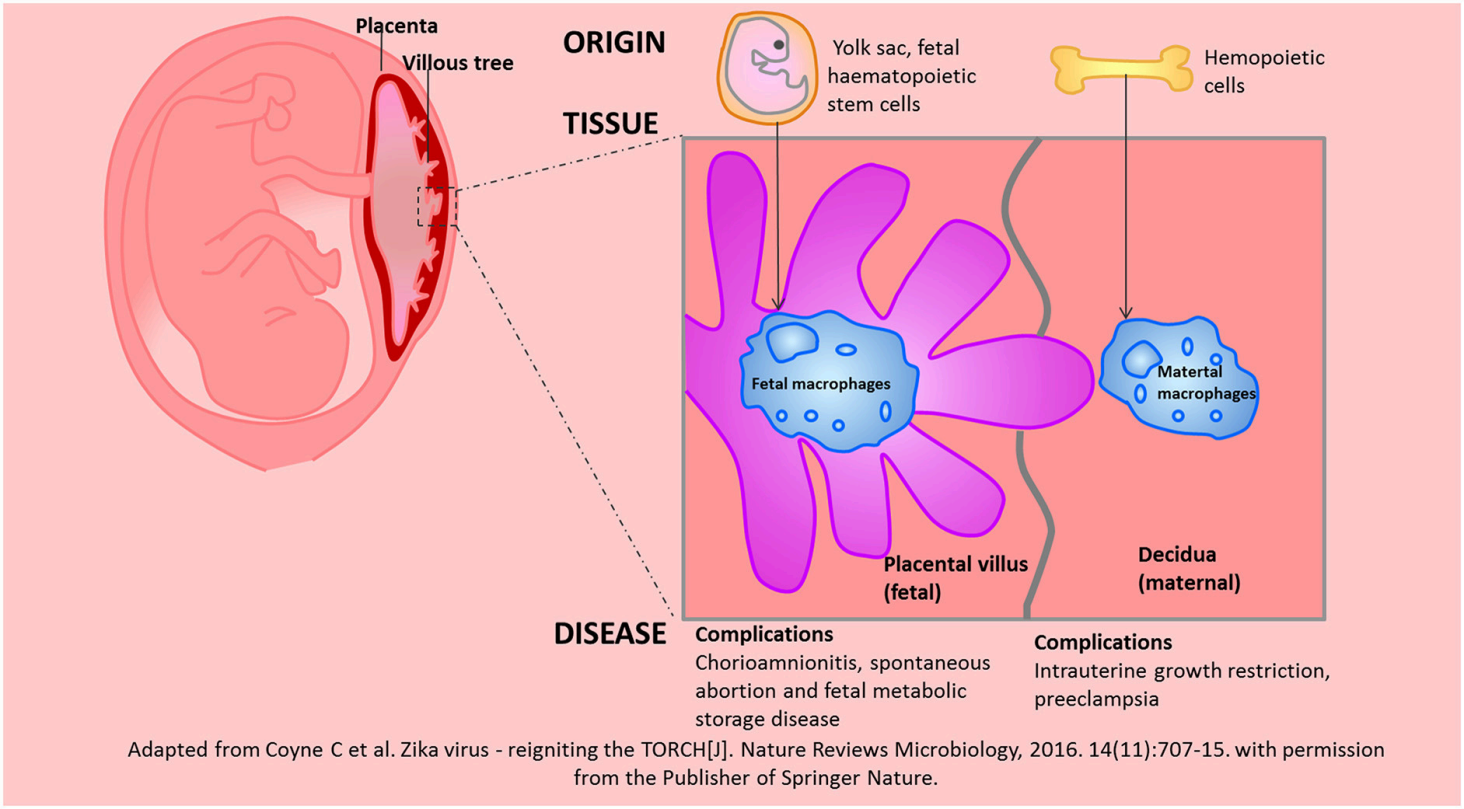
The similarities and differences between Hofbauer cells and maternal macrophages in the placenta were depicted according to the origin, resident tissue and related diseases. Adapted from Coyne et al. (139) with permission from the publisher of Springer Nature.

Hofbauer cells have been found to play critical roles in maternal-fetal immune tolerance since the 1990s. Bockle found that Hofbauer cells highly express CD163 and DC-SIGN/CD209 in the term placenta. Thus, Hofbauer cells have been suggested to be M2 macrophages (85). However, CD163, DC-SIGN, and CD206 (M2 markers) were not clearly detected in the term placenta in the study by Joerink et al. whereas CX3CR1, IL-7R or CCR7 (M1 markers) were observed in the term placenta (86). Studies have also shown that Hofbauer cells are positive for CD209 (M2a marker), CD86 (M2b marker), HLA-DR (M2a/M2b marker), CD206 (M2a/M2c), and CD14 (M2c marker) (82). Recently, Kim SY et al. demonstrated that the genes encoding markers of M1 macrophages, such as TLR9, IL1B, IL12RB2, CD48, and FGR, were hypermethylated in Hofbauer cells, whereas the genes encoding markers of M2 macrophages, such as CCL2, CCL13, CCL14, CD209 and A2M, were hypomethylated in Hofbauer cells (87). Hofbauer cells may promote placental angiogenesis, chorionic villus growth, and stromal fluid balance, absorb immune complexes and function as antigen presenting cells (88). Perturbed Hofbauer cells function is a common occurrence in chorioamnionitis, spontaneous abortion and fetal metabolic storage disease. Although both of Hofbauer cells and maternal macrophages are predominantly M2 phenotypes, they have different origins, resident tissues, biological functions, and associated complications. We depicted these differences in the Figure 4.

## The Roles of Macrophages in Pregnancy Complications

### The Roles of Macrophages in Miscarriage

Miscarriage, especially recurrent spontaneous miscarriage, is an immune-related reproductive disorder (89). The WHO defines 3 or more consecutive miscarriages before the 20th week of gestation as recurrent miscarriage (90). The definition from American College of Obstetricians and Gynecologists is “just 2 consecutive miscarriages” (91). The study on human decidual tissues reported that the number of CD68^+^ macrophages is higher in recurrent miscarriage patients than in artificial abortions patients (92). Similarly, it has also been observed that macrophage depletion could rescue CpG ODN (CpG-Oligodeoxynucleotides)-induced fetal resorption in the CBA/J x DBA/2 mice model (93). Cathepsins belong to the family of lysosomal cysteine proteases and play important roles in the degradation of matrix molecules and intracellular proteolysis. It has been shown that the expression of cathepsin B, D, H was upregulated and cathepsin E was downregulated in the decidual tissues of spontaneous miscarriage patients compared to normal pregnancy patients (94). Cathepsin-deficient (CatE^−/−^) mice were fertile, but the litter sizes were smaller than those of wild-type mice (95). The percentage of FasL^+^/CD68^+^ cells is increased in spontaneous abortion patients compared to normal pregnancy subjects. These results implied that Fas/FasL mediated apoptosis of macrophages involved in the occurrence of abortion (6).

Macrophages are skewed toward the M1 phenotypes in spontaneous abortion (96). The ratio of M1/M2 on day 16 was 3.9–4.2 in the abortion-prone mice, while the number was 1.2–1.6 on both day 12 or 16 in the non-abortion-prone mice (97). In decidual macrophages from patients with spontaneous abortion, the expression levels of CD80, IL-12, and IL-13 were increased, while the expression levels of CD163, CD206, IL-10, and ARG-1 were decreased (98, 99). PPARγ is essential for the differentiation of alternatively activated (M2) macrophages (100). PPARγ was significantly downregulated in placental tissues from women with recurrent miscarriages. This implies that downregulation of PPARγ expression may skew macrophages to the M1 phenotype and lead to miscarriages (101). IL-33, a member of the IL-1 family, induced the proliferation of cytotrophoblasts (CTB) and triggered the migration of EVT by interacting with the IL-33 ligand ST2L (102). Dysregulation of the IL-33/ST2 signaling pathway may skew normal pregnancy-derived dMϕs and U937 cells into the M1 phenotype (103). Meng et al. observed that the levels of RANKL/RANK were reduced in villi and decidua from miscarriage patients compared to those from normal pregnancy patients. Downregulation of nuclear factor-κ B ligand (RANKL) caused murine fetal loss. The abnormal expression of RANKL may switch macrophages into M1 phenotype through the Akt/STAT6-Jmjd3/IRF4 signaling pathway (104) (Table 2).

**Table 2:** The roles of macrophages in miscarriage.

**Samples**	**Mechanisms**	**References**
Human	Cathepsin B, D, H are upregulated and cathepsin E is downregulated in the decidual tissues;	(94)
Mice	The litter sizes of *CatE*^−/−^ mice are smaller;	(95)
Mice	The Fas/FasL mediated apoptosis is increased;	(6)
Human	CD80, IL-12, IL-13 are increased, while CD163, CD206, IL-10, ARG-1 are decreased;	(98, 99)
Human	Reduced the expression of PPARγ may skew macrophages to the M1 phenotype;	(101)
Human and U937 cell line	Dysregulation of the IL-33/ST2 signaling pathway may skew macrophages into the M1 phenotype;	(103)
Human and mice	The decreased expression of RANKL could switch macrophages into M1 phenotype through the Akt/STAT6-Jmjd3/IRF4 signaling pathway.	(104)

### The Role of Macrophages in Preeclampsia

Preeclampsia, a pregnancy-specific disorder characterized by hypertension in combination with proteinuria, occurs at 20 weeks after gestation (105). With a prevalence of 6–8% of pregnancies, preeclampsia is a major cause of maternal and fetal morbidity and mortality (106). The pathophysiological mechanism of preeclampsia has not been elucidated in detail; however, preeclampsia is associated with impaired spiral artery remodeling and with changes in the numbers of trophoblasts and immune cells in the placenta. Decreased numbers of decidual macrophages were reported in patients with preeclampsia (107, 108). However, various studies have found increased numbers of macrophages in the placenta of patients with preeclampsia (109–111). Conflicting findings across studies may be due to the use of different cell makers or methodologies and different locations of the placenta being studied.

The numbers of macrophages were changed in preeclamptic patients, and the polarization states of macrophages were different in preeclamptic patients compared to normal pregnancy patients. A decreased number of M2 macrophages and an increased number of M1 macrophages in the placenta may be related to preeclampsia (112, 113). This finding is in concordance with an increase in pro-inflammatory cytokines (such as TNF-α, IL-6, and IL-8) and a decrease in anti-inflammatory cytokines (such as IL-10) in the placenta of preeclamptic patients (114). Various tissue-derived mesenchymal stem cells (MSCs) regulate the polarization of macrophages (115–118). Human placental MSCs can shift macrophages from an M1-like to an M2-like phenotype (115). In line with this finding, human amniotic MSCs have anti-inflammatory properties and skew macrophages toward a M2 phenotype (119). In patients with preeclampsia, TGF-β3 in decidua could promote the expression of miR-494 in dMSCs and downregulate the effect of dMSCs switching the macrophages toward M2 phonetype (117).

The altered amount and polarization phenotypes of uterine macrophages may account for the defective trophoblast invasion and spiral artery remodeling observed in preeclampsia. Aberrantly activated macrophages are capable of producing various molecules (such as TNF-α and IFN-γ) that may affect trophoblast invasion by the reconstitution of the extracellular matrix (ECM) (120, 121). Lockwood CJ et al. demonstrated that TNF- α bound to TNF-αR and caused the increased expression of MMP-1, MMP-3, and MMP-9 by activating p38 MAPK phosphorylation in decidual cells, whereas IFN-γ bound to IFN-γR and blocked TNF-α-induced p38 phosphorylation to protect against MMP-mediated ECM degradation (122). There is evidence that placental apoptosis was increased in preeclampsia. The elevated levels of pro-inflammatory cytokines secreted by aberrantly activated macrophages (such as TNF-α and IFN-γ) may increase the sensitivity of trophoblast cells to apoptosis and restrict trophoblast invasion (111, 123). TNF-α and IFN-γ have been shown to increase the expression of the pro-apoptotic factor X-linked inhibitor of apoptosis (XIAP) in trophoblast cells and initiate the caspase-dependent pathway (124). Deficient spiral artery remodeling is hypothesized to account for the major pathogenesis of early-onset preeclampsia (125). It has been proven that macrophages were associated with impaired spiral artery remodeling in patients with preeclampsia (126). In addition, it has been speculated that ATP-induced activated macrophages may prevent spiral artery remodeling in preeclampsia based on the fact that more activated macrophages were observed in the mesometrial triangle of ATP-infused rats, and spiral artery remodeling in the rat mesometrial triangle was impaired (127) (Table 3).

**Table 3:** The roles of macrophages in preeclampsia.

**Samples**	**Mechanisms**	**References**
Human	TNF-α, IL-6 and IL-8 are increased, IL-10 is decreased;	(114)
Human	Amniotic MSCs could skew macrophages toward a M2 phenotype;	(119)
Human	TGF-β3 promotes the expression of miR-494 in dMSCs and downregulates the effect of dMSCs switching the macrophages toward a M2 phonetype;	(117)
Human	Macrophages produce TNF-α and IFN-γ to affect trophoblast invasion by the reconstitution of the ECM;	(120, 121)
Human	TNF-α increases the expression of MMP-1, 3, 9 by activating p38 MAPK phosphorylation in decidual cells, whereas IFN-γ blocked TNF-α-induced p38 phosphorylation to protect against MMP-mediated ECM degradation.	(122)
Human	TNF-α and IFN-γ increase the sensitivity of trophoblast cells to apoptosis;	(111, 123)
Human	TNF-α and IFN-γ increase the XIAP expression and initiate the caspase-dependent pathway;	(124)
Rats	More activated macrophages and impaired spiral artery remodeling are observed in the mesometrial triangle of the ATP-infused rats.	(127)

### The Role of Macrophages in Preterm Birth

Preterm birth, the birth of a baby at fewer than 37 weeks of gestational age (128), is the most common cause of death among infants worldwide (129). Inflammation has been considered to be associated with preterm birth (130). An increased number of macrophages have been observed in the cervix of women in preterm labor (131). Studies have been reported that the depletion of F4/80^+^ macrophages could rescue the CpG-induced preterm birth of mice to term (132). Studies have shown that macrophages induce the release of MMPs and collagen degradation in the cervix of mice that deliver at preterm (133). The interaction of C5a, a chemotactic factor and activator of macrophages, with C5aR is necessary for macrophages to release MMP-9 and to be involved in the cervical remodeling process. Progesterone was reported to decrease the expression of C5aR and inhibit preterm birth in mice (8). Human chorionic gonadotropin (HCG) has been confirmed to stimulate progesterone production (134), thus having anti-infammatory capacity and preventing endotoxin-induced preterm birth in mice. The numbers of M1 macrophages in decidual tissue from spontaneous preterm labor patients were much greater than those in term without labor patients. Studies have also proposed that both M1 (CD11c^+^) and M2 (CD206) macrophages participate in preterm birth since the expression levels of both pro-inflammatory (IL-6, IFN-γ) and anti-inflammatory cytokines (IL-10) were significantly increased in the uterus of PGN+poly (I:C)-treated preterm labor mice (135). The Notch signaling pathway has been considered to promote the M1 polarization of macrophages (136). During inflammation-induced preterm labor in mice, decidual macrophages were polarized into the M1 subtype by activating the Notch signaling pathway, which could be blocked by a2V (137). Xu Y et al. demonstrated that decidual M1-like macrophages were associated with spontaneous preterm labor patients. The activation of PPARγ via rosiglitazone could attenuate the macrophage-mediated pro-inflammatory response and prevent preterm birth in mice (45) (Table 4).

**Table 4:** The roles of macrophages in preterm birth.

**Samples**	**Mechanisms**	**References**
Mice	Macrophages induce the release of MMPs and collagen degradation in the cervix;	(133)
Mice	Progesterone decreases the expression of C5aR and then inhibits the release of MMP-9 to protect against the PTL;	(8)
Mice	HCG stimulates the production of progesterone and prevents endotoxin-induced PTL;	(134)
Mice	Macrophages polarize into the M1 subtype by activating the Notch signaling pathway, which could be blocked by a2V;	(137)
Human and mice	The activation of PPARγ attenuates the macrophage-mediated pro-inflammatory response and prevents PTL.	(45)

## Conclusions

Altogether, this review summarized the current knowledge of the polarization of macrophages and their regulatory mechanisms at different stages of pregnancy, as well as the roles of these cells in pathological processes. Although current evidence provides a compelling argument that macrophages are important in pregnancy, our understanding of the roles and mechanisms of macrophages in pregnancy is still rudimentary. Since macrophages exhibit functional plasticity, they may be ideal targets for therapeutic manipulation during pathological pregnancy. Additional studies are needed to better define the functions and mechanisms of various macrophage subsets in both normal and pathological pregnancy.

## Author Contributions

All authors listed have made a substantial, direct and intellectual contribution to the work, and approved it for publication.

### Conflict of Interest Statement

The authors declare that the research was conducted in the absence of any commercial or financial relationships that could be construed as a potential conflict of interest.
